# Nasal Administration of a Nanoemulsion Based on Methyl Ferulate and Eugenol Encapsulated in Chitosan Oleate: Uptake Studies in the Central Nervous System

**DOI:** 10.3390/pharmaceutics17030367

**Published:** 2025-03-13

**Authors:** Giada Botti, Laura Catenacci, Alessandro Dalpiaz, Luca Randi, Maria Cristina Bonferoni, Sara Perteghella, Sarah Beggiato, Luca Ferraro, Barbara Pavan, Milena Sorrenti

**Affiliations:** 1 Department of Chemical, Pharmaceutical and Agricultural Sciences, Via L. Borsari 46, I-44121 Ferrara, Italy; bttgdi@unife.it; 2 Department of Drug Sciences, University of Pavia, Viale Taramelli 12, I-27100 Pavia, Italy; laura.catenacci@unipv.it (L.C.); luca.randi@unipv.it (L.R.); mariacristina.bonferoni@unipv.it (M.C.B.); sara.perteghella@unipv.it (S.P.); milena.sorrenti@unipv.it (M.S.); 3 Department of Life Sciences and Biotechnology and LTTA Center, University of Ferrara, Via L. Borsari 46, I-44121 Ferrara, Italy; bggsrh@unife.it (S.B.); frl@unife.it (L.F.); 4 Department of Neuroscience and Rehabilitation—Section of Physiology, University of Ferrara, Via L. Borsari 46, I-44121 Ferrara, Italy; pvnbbr@unife.it

**Keywords:** eugenol, ferulic acid, methyl ferulate, chitosan oleate, nanoemulsion, nasal administration, brain targeting

## Abstract

**Background/Objectives**: The phytochemicals ferulic acid (Fer) and eugenol display neuroprotective effects for their anti-oxidative properties; moreover, eugenol can induce dopamine (DA) release from dopaminergic neuronal cells. However, poor bioavailability and/or fast elimination rate limit their clinical benefits. We therefore propose a new nasal formulation based on a nanoemulsion (NE) for the jointed brain-targeting of eugenol and methyl ferulate (Fer-Me, i.e., a Fer-lipidized derivative maintaining the parent compound anti-oxidative properties). NE was obtained using chitosan oleate, a surfactant combining mucoadhesive and absorption-enhancing properties with stabilizing effects on the dispersion of eugenol, used as a Fer-Me vehicle. **Methods**: The nasal formulation was obtained by spontaneous emulsification processes; cell viability and uptake studies were performed on an in vitro model of respiratory mucosa (RPMI 2650 cells). After intravenous and nasal administrations, the pharmacokinetic profiles of eugenol and Fer-Me in rats’ bloodstreams and cerebrospinal fluid (CSF) were analyzed via HPLC-UV analysis. **Results**: The NE dispersed-phase mean diameter was 249.22 ± 32.78 nm; Fer-Me and eugenol loading in NE was about 1 and 2 mg/mL, respectively. NE increased the uptake of loaded compounds by mucosal cells. Following intravenous administration, the Fer-Me plasma half-life was 10.08 ± 0.37 min, and a negligible ability of the compound to permeate in the CSF, compared to eugenol, was observed. NE nasal administration allowed us to sensibly increase the Fer-Me brain-targeting and prolong the eugenol permanence in the CSF. **Conclusions**: This nasal formulation appears promising to overcome Fer and eugenol pharmacokinetic issues. The possible translational relevance of the present findings is discussed.

## 1. Introduction

Phytochemicals are natural bioactive compounds produced by plants, generally for their own protection. Among these compounds, polyphenols and essential oils are particularly relevant mainly due to their recognized anti-inflammatory and antioxidative properties [1,2], leading to their proposed role as therapeutic agents for neurodegenerative disease prevention/treatment [3,4,5,6]. In particular, the polyphenol ferulic acid (Fer) displays protective effects against neurodegenerative disorders, including Parkinson’s disease (PD) [5], because of its interesting potential, which includes anti-inflammatory and antioxidant properties, mainly related to the inhibition of reactive oxygen species (ROS) production [7,8]. The neuroprotective properties of Fer may be further exploited by intranasally administrating its methyl ester derivative methyl ferulate (Fer-Me). In fact, we recently demonstrated that Fer-Me retains the intrinsic anti-inflammatory and antioxidant activities of Fer, and can act as a Fer prodrug, also improving its encapsulation in hydrophobic particulate systems designed for nasal administration [9]. On the other hand, eugenol, a main component of essential oils, is known for its anti-inflammatory profile as well as its efficacy against oxidative stress through the inhibition of enzymes and oxidative processes [10]. Moreover, very recently, we have evidenced that eugenol is efficacious in stimulating in vitro neuronal cell viability and inducing a time- and dose-dependent release of dopamine (DA) [11]. It could be therefore hypothesized that the co-administration of Fer and eugenol, by combining the neuroprotective properties of both compounds with the DA-releasing effects of eugenol, might represent a promising strategy for the prevention and/or treatment of PD, i.e., a neurodegenerative disease associated with the loss of substantia nigra (SNc) dopaminergic neurons [12,13,14]. However, the poor oral bioavailability or the rapid metabolism, which generally minimize the beneficial effects of phytochemicals [15,16] including polyphenols and essential oils, might hinder the development of Fer/eugenol therapeutic strategies for PD. In the attempt to overcome this issue, we propose a new nano-emulsified formulation based on eugenol where Fer-Me was dispersed, stabilized by chitosan oleate salt (CS-OA) used as a surfactant [17], hereinafter referred to as CS-OA NE. Fer-Me was chosen considering its better aptitude to be loaded in lipophilic dispersed systems than Fer [9]. In this formulative approach, designed for nasal administration, eugenol also appears as a promising vehicle for Fer-Me, able to support its neuroprotective aptitude in the central nervous system (CNS) [10] and inducing, at the same time, DA release [11]. The presence of CS-OA, obtained by ionic interaction between chitosan HCl (CS) and oleic acid (OA), combines the advantages of CS as a mucoadhesive and absorption enhancer with the stabilization effect of the salt. This amphiphilic polymer, in fact, acts as a stabilizer of nanoemulsions (NEs) obtained by spontaneous emulsification with good protection of the volatile essential oil encapsulated [17,18,19].

In the present study we mainly describe the design and the characterization of this novel nasal formulation for the jointed administration of Fer-Me and eugenol. Furthermore, based on the encouraging results from the in vitro cell studies, in vivo pilot experiments aimed at evaluating the ability of this innovative formulation to induce eugenol and Fer-Me CNS targeting, via the nose-to-brain route, have been performed. To this aim, the pharmacokinetic profiles of eugenol and Fer-Me in the bloodstream and cerebrospinal fluid (CSF), following their intravenous and nasal administration, have been compared. The results suggest that the CS-OA NE nasal formulation is suitable to induce eugenol/Fer-Me brain targeting, thus representing an interesting formulation to evaluate, in further study, the potential therapeutic properties of the two-compound combination for PD prevention/treatment.

## 2. Materials and Methods

### 2.1. Materials

Eugenol, dimethyl sulfoxide (DMSO), benznidazole (BNZ), acetic acid glacial, and bovine serum albumin (BSA) were obtained from Merck Life Sciences Srl (Milan, Italy). Methyl ferulate (Fer-Me) and methyl caffeinate (Caf-Me) were synthesized as previously described [9,20]. Stock solutions of Fer-Me and eugenol, each dissolved in DMSO (0.05 M final concentrations), were stored at −20 °C until their use for in vivo or in vitro studies. Chitosan HCl (CS) (low molecular weight, 80–95% degree of deacetylation) was obtained from Sigma Aldrich (Milan, Italy) and oleic acid (OA) was obtained from Fluka (Milan, Italy).

Acetonitrile (CH_3_CN), ethanol (EtOH), methanol (MeOH), acetic acid, formic acid, sodium acetate, and water (H_2_O) were of high-performance liquid chromatography (HPLC) grade from Carlo Erba Reagents S.A.S. (Milan, Italy). Advanced Minimum Essential Medium (A-MEM), L-glutamine, fetal bovine serum (FBS), penicillin, streptomycin, trypsin-EDTA, and Dulbecco’s phosphate buffered saline (DPBS) were furnished by Thermo-Fisher Scientific (Milan, Italy). Thermo-Fisher Scientific (Milan, Italy) and Biosigma (Venice, Italy) furnished the cell culture vessels. RPMI 2650 cell line (CCL-30) [21,22] was purchased from American Type Culture Collection (ATCC, Cat. number: CCL-30™, Manassas, VA, USA).

### 2.2. Formulation and Characterization of CS-OA NE

#### 2.2.1. Preparation of Nanoemulsions (Encapsulation of Fer-Me and Eugenol in CS-OA Micelles)

To promote the self-assembly of CS-OA micelles during NE preparation, CS-OA was synthesized directly within the system as previously reported [19]. A 1:1 molar ratio of CS to OA was consistently used, according to a calculation based on the theoretical availability of free amino groups on CS [23].

Eugenol was used in such quantities as to obtain a 1:1 weight ratio with CS-OA, a proportion previously observed to yield NEs with optimal size. Preliminary evaluation of Fer-Me solubilization in eugenol was performed by adding increasing volumes of the oil to a weighed amount of Fer-Me. The organic phase was prepared by dissolving Fer-Me in eugenol at 30% *w*/*v* and adding OA (the proper amount to reach the molar ratio 1:1 between OA and CS); the obtained solution was mixed with EtOH. Spontaneous emulsification was performed as previously reported [17]; briefly, the organic phase was gradually added dropwise into a 0.2% *w*/*v* aqueous solution of CS maintained under continuous magnetic stirring. EtOH was then allowed to evaporate overnight under gentle stirring. Finally, the sample was sonicated for 15 min at 20% amplitude with a pulse cycle (10 s on/10 s off), using a Fisherbrand Ultrasonic Liquid Processor (model FB-505, output frequency of 20 kHz).

The reproducibility of the production process was determined by preparing and characterizing six batches of NEs stored at 4 °C until use.

#### 2.2.2. FT-IR Analysis

Attenuated total reflectance–Fourier Transform Infrared Spectroscopy (FT-IR) analysis in the mid-IR range (650–4000 cm^−1^) was carried out using a Spectrum One FT-IR spectrophotometer (PerkinElmer, Monza, Italy) equipped with a MIRacle™ ATR accessory (PIKE Technologies, Madison, WI, USA). The measurements were conducted with 64 scans at a resolution of 4 cm^−1^;. The sample was positioned onto a Zinc Selenide (ZnSe) ATR crystal and the spectra were acquired in transmittance mode. The FT-IR analysis was conducted to assess the interactions between the two components. The analyses were carried out on the pure components, eugenol, Fer-Me, and Fer-Me solubilized in eugenol (30% *w*/*v*), indicated as a physical mixture (PM). For each sample, the spectra were collected at least in triplicate to evaluate reproducibility.

#### 2.2.3. Particle Size Characterization

The mean size and the polydispersity index (PDI) of the dispersed phase were evaluated on six different batches of NEs using Photon Correlation Spectroscopy (PCS) with an N5 Submicron Particle Size Analyzer (Beckman Coulter, Milan, Italy) equipped with a 25 mW Helium-Neon Laser (632.8 nm). PCS measurements were performed with a 90° detection angle for the scattered light, at 25 °C. Before analysis, the samples were diluted in 0.22 μm filtered, bi-distilled water. Three different batches of NEs were evaluated for size stability after 1, 2, and 4 weeks of storage at 4 °C.

#### 2.2.4. Transmission Electron Microscopy (TEM)

NE morphology was studied by a TEM microscope (JEOL JEM-1200EX-II, Milan, Italy). A 300-mesh copper grid was placed on an NE droplet for 1 min; thereafter, the sample was stained by depositing the grid on a drop of 2% uranyl acetate solution for 2 min and the excess solution was wicked away with a wet filter paper.

#### 2.2.5. HPLC Analysis

The eugenol and Fer-Me quantification was performed using an HPLC apparatus, coupled with a UV-Vis Variable Wavelength Detector equipped with a deuterium lamp with a wavelength accuracy of ±1 nm (Agilent 1100 Series, model G1314A, Santa Clara, CA, USA). The mobile phase was constituted by a mixture of CH_3_CN and H_2_O 38:62 (*v*/*v*) acidified with formic acid at pH 3.25, the flow rate was maintained at 1 mL/min, and separations were performed at room temperature on a 150 mm × 4.6 mm, 3 m i.d. SUPELCOSIL^TM^ LC-18 endcapped silica column (Supelco^®^, Merck, Milan, Italy). Eugenol was detected at a wavelength of 210 nm, while Fer-Me was detected at 320 nm. The injection volume was 20 µL for each sample. The retention times recorded for eugenol and Fer-Me were 4.6 min and 7.6 min, respectively. Their quantification was performed by associating the peak area with a standard curve established over the range 1–100 µg/mL (4.8–480 µM for Fer-Me and 6.1–610 µM for eugenol); the calibration curve was linear both for Fer-Me (*n* = 3, *r*^2^ = 0.999, *p* < 0.001) and eugenol (*n* = 3, *r*^2^ = 0.998, *p* < 0.001). An ANOVA was performed to assess the feasibility of averaging the peak area values across three calibration curves for both the analytes. These analyses, conducted to evaluate the reproducibility and robustness of the method, yielded *p* = 0.999, confirming no statistically significant differences between the data points and that the curves are equivalent and can be combined into a single average calibration curve. Both standard curve and NE samples were injected after dilution in a mixture of mobile phase and acetate buffer (5 mM); every analysis on each sample was done in triplicate.

#### 2.2.6. Process Yield and Encapsulation Efficiency (EE)

The yield of the process was verified by quantifying by HPLC analysis Fer-Me and eugenol loaded into the CS-OA NEs immediately after preparation and after 1, 2, and 4 weeks of storage at 4 °C. Results were obtained by analyzing four batches of NEs, which were used to calculate the yield percentage (% Yield) and final concentration (µg/100 µL of NE). % Yield was calculated by Equation (1):

(1) $$\%\;\mathrm{Yield}=\frac{\;\mathrm{total}\;\mathrm{drug}\;\mathrm{amount}\;\mathrm{in}\;\mathrm{CS}-\mathrm{OA}\;\mathrm{NE}}{\mathrm{theoretical}\;\mathrm{drug}\;\mathrm{amount}}\;\times \;100$$

The EE% was evaluated using the ultrafiltration method [24] with PES membrane (50K MWCO, Thermo Scientific, Waltham, Massachusetts, USA) at 4000 *g* for 10 min to separate the dispersed phase from the external one. Fer-Me and eugenol were quantified by the HPLC method into the external aqueous phase, passing through the membrane as permeate.

EE% was calculated by Equation (2):

(2) $$\mathrm{EE}\%=\frac{\;\mathrm{total}\;\mathrm{drug}\;\mathrm{amount}\;\mathrm{in}\;\mathrm{CS}-\mathrm{OA}\;\mathrm{NE}\;-\;\mathrm{drug}\;\mathrm{amount}\;\mathrm{in}\;\mathrm{external}\;\mathrm{phase}}{\mathrm{total}\;\mathrm{drug}\;\mathrm{amount}\;\mathrm{in}\;\mathrm{CS}-\mathrm{OA}\;\mathrm{NE}}\;\times \;100$$

#### 2.2.7. Drug Release

The in vitro release profiles of Fer-Me and eugenol from CS-OA NE were assessed using dialysis tubing (MWCO 12–14 kDa), which contained 2 mL of the formulation. The membrane was immersed in 50 mL of PBS (pH 6.2) at 32 ± 2 °C. The release medium was kept under constant magnetic stirring. Aliquots (1 mL) were withdrawn at predetermined intervals ranging from 15 min to 4 h and replaced with an equal volume of fresh PBS. The Fer-Me and eugenol released were quantified using the HPLC method.

#### 2.2.8. Mucoadhesion Test

The mucoadhesive behavior was assessed by analysis of the zeta potential [25], evaluating the interaction between mucin and CS. CS-OA NE was diluted 1:10 with MilliQ water and the zeta potential of the sample as such, mixed 1:1 (*v*/*v*) for 30 min with 0.1% and 0.5% (*w*/*v*) of gastric porcine mucin (Sigma Aldrich, Milan, Italy) was measured.

Zeta potential measurements were carried out using a Litesizer 500 (Antoon Paar, Turin, Italy); each measurement was performed in triplicate (*n* = 3).

### 2.3. In Vitro Cytotoxicity and Uptake Studies in RPMI 2650 Cells of CS-OA NE

#### 2.3.1. RPMI 2650 Cell Culture

RPMI 2650 cells were used to evaluate in vitro the possible CS-OA NE cytotoxicity as well as Fer-Me and eugenol cell uptake. This cell line derives from an anaplastic squamous cell carcinoma of the human nasal septum and, therefore, exhibits epithelial morphology [21,22], allowing for the constitution of an in vitro model of respiratory mucosa.

RPMI 2650 cells were cultured on 75 cm^2^ polystyrene cell culture flasks in Advanced Minimum Essential Medium (A-MEM), which was supplemented with 10% fetal bovine serum (FBS), 2 mM L-glutamine, 100 IU/mL penicillin, and 100 μg/mL streptomycin. The cells were maintained in a humidified atmosphere containing 5% CO_2_ at 37 °C. Cells were split twice using trypsin-EDTA at 37 °C before being seeded and allowed to grow to approximately 90% confluence for 3-(4,5-dimethylthiazol-2-yl)-2,5-diphenyltetrazolium (MTT) assays and uptake studies. Cell counting was performed using Scepter™ 2.0 handheld automated cell counter (Merck Millipore, Milan, Italy).

#### 2.3.2. MTT Assay for Evaluation of Fer-Me and Eugenol Toxicity

RPMI 2650 cells were seeded in a 96-well plate at a density of 3 × 10^4^ cells/well containing 0.2 mL of growth A-MEM. The potential cytotoxicity of Fer-Me and eugenol was assayed by MTT test. To this purpose, cells were incubated for 1 h under standard conditions (37 °C and 5% CO_2_) with increasing dilutions (1:2, 1:20, 1:50, 1:100, 1:500, 1:1000 *v*/*v*) of CS-OA NE or a solution containing Fer-Me and eugenol as pure compounds at concentrations comparable to those of CS-OA NE (approximated Fer-Me = 5 mM; eugenol = 6 mM). Following the incubation period, sample media were discarded and 0.2 mL of MTT solution (0.5 mg/mL) in growth A-MEM was added to each well (3 h; 37 °C; 5% CO_2_), to allow the conversion of the tetrazolium yellow salt by metabolically active cells to chromogenic purple formazan crystals. The crystals were then solubilized by shaking for 1 h (37 °C) in DMSO (0.1 mL/well) and the absorbance of each well was measured at 570 nm using a microtiter plate reader (NB-12-0035 Microplate Reader, NeoBiotech, distributed by CliniSciences, Rome, Italy). The results were obtained as an MTT reduction percentage in RPMI 2650 cells treated with culture medium (100% viability as negative control) compared to those treated with test compounds. The values are reported as the mean from three independent incubation experiments, each performed in duplicate (*n* = 6).

#### 2.3.3. Time Course of Fer-Me and Eugenol Uptake in RPMI 2650 Cells

RPMI 2650 cells were seeded in a 12-well plate at a density of 8 × 10^4^ cells/well containing 2 mL of culture A-MEM. The potential uptake of Fer-Me and eugenol loaded in the NE by RPMI 2650 cells was evaluated at the lower non-toxic dilution detected by MTT assays (1:20 *v*/*v*). The corresponding solution of pure compounds (approximated to 250 µM and 600 µM concentrations of Fer-Me and eugenol, respectively), and raw Fer-Me (corresponding to a 250 µM solution) added directly to the wells as powder, were used as comparison. In particular, 2 mL of diluted CS-OA NE or the corresponding solution of pure compounds was added to the test wells, and the plate was incubated for pre-determined interval times (10, 30, and 60 min) in a humidified 5% CO_2_ atmosphere at 37 °C. As for raw Fer-Me incubation, 0.1 mg was accurately weighed (analytical balance Sartorius CP 225D), added to each well containing 2 mL of complete A-MEM, and incubated at 37 °C in an orbital shaker for the same pre-determined interval times above indicated. All media were removed at the end of the incubation. The well-adhering cells were first rinsed thrice with DPBS (2 mL), then lysed by adding 0.2 mL of bi-distilled water (about 4 °C), and kept at −80 °C for at least 30 min. After thawing, the lysate was centrifuged twice (16,000× *g* for 10 min) and the supernatant was immediately injected into the HPLC apparatus (20 μL) to quantify Fer-Me and eugenol (see below). The final number of cells was evaluated on untreated wells after their trypsinization and counted using the Scepter^TM^ 2.0 automated cell counter (Merck Millipore, Milan, Italy). Each reported value represents the mean of three independent incubation experiments expressed as nanomoles of compounds for 10^6^ cells. The uptake efficiencies were calculated as the compound amount quantified in cell lysate 10 min after the onset of the incubation compared to the initial compound amount incubated in cell media (%). The values are represented as the percentage of drugs taken up by the cells. CS-OA NE uptake was compared to the corresponding values obtained following the incubation with pure compounds or suspension of Fer-Me [26].

The quantification of eugenol and Fer-Me was performed by using a modular HPLC system composed of a pump (model LC-40D), DAD detector (model SPD-M40, Shimadzu, Kyoto, Japan), as well as an injection valve with a 20 μL sample loop (model 7725; Rheodyne, IDEX, Torrance, CA, USA). For the elution process, a 5 μm Hypersil BDS C-18 column (150 mm × 4.6 mm i.d.; Thermo Fisher Scientific SpA Italia Srl, Milan, Italy) was used, along with a guard column packed with the same Hypersil material. The mobile phase was isocratic and composed of a mixture of H_2_O and CH_3_CN (38:62 *v*/*v*), with a flow rate set at 1 mL/min. To assess the absorbance of eugenol and Fer-Me, chromatograms were displayed at wavelengths of 210 nm and 320 nm, respectively. Fer-Me and eugenol retention times were found to be 6.2 min and 11.4 min, respectively. LabSolutions Software (version 5.110, Windows 10, Shimadzu, Kyoto, Japan) was used for data acquisition and processing. The precision of the chromatographic analysis was evaluated through repeated analysis (*n* = 6) of the same sample solution containing each compound singularly at 10 μM concentration (2.08 µg/mL for Fer-Me and 1.64 µg/mL for eugenol) dissolved in H_2_O or in a mixture of H_2_O and CH_3_CN (25:75 *v*/*v*) and was represented by relative standard deviation (RSD) values ranging from 0.85 to 0.92 for all the compounds analyzed. Calibration curves of peak areas as a function of analyte concentrations were created for the mixture of compounds dissolved in H_2_O with concentrations ranging from 0.3 to 100 μM (0.06 to 20.82 μg/mL for Fer-Me and 0.05 to 16.43 μg/mL for eugenol), showing a linear relationship (*n* = 6, *r* ≥ 0.994, *p* < 0.0001).

### 2.4. In Vivo Administration

Fer-Me and eugenol were nasally co-administered as loaded in CS-OA NE to evaluate their concentration–time profiles in male Sprague-Dawley rats’ (200–250 g body weight; Charles River laboratories, Calco, Italy) bloodstream and CSF. The results were compared with those obtained by the rat intravenous administrations of Fer-Me (10 mg/kg; present study), and 20 mg/kg of eugenol [11]. The sample size (4 rats/group) has been chosen based on our previous studies [11,20,27,28,29]. The experimental procedures were approved by the Italian Ministry of Health and were performed in accordance with the European Communities Council Directive of September 2010 (2010/63/EU). According to the ARRIVE guidelines, all possible efforts were made to minimize animal pain and discomfort and to reduce the number of experimental subjects.

#### 2.4.1. Intravenous Administration of Fer-Me

Fer-Me solution (2.5 mg/mL final concentration, 15% *v*/*v* EtOH content) was prepared by first dissolving Fer-Me in EtOH and then adding this latter solution to a saline solution (0.9% NaCl). Fer-Me (10 mg/kg dose) was administered intravenously via femoral infusion of 2.5 mg/mL Fer-Me solution (rate = 0.2 mL/min; 5 min) to a group (*n* = 4) of rats, each weighing approximately 250 g, which had been fasted for 12 h and anesthetized during the experimental procedure.

Blood (100 μL) and CSF (30 μL) samples were collected at specific time points after the end of infusion and immediately the samples of blood (*n* = 4 for time point) were quenched in ice-cold H_2_O (500 μL). Subsequently, sulfosalicylic acid (10%, 50 μL) and the internal standard (100 μM Caf-Me dissolved in a 50:50 *v*/*v* mixture of MeOH and H_2_O, 50 μL) were added. The aqueous phase was then subjected to a double extraction with 1 mL of H_2_O-saturated ethyl acetate. Following the extraction, the samples were centrifuged at 13500× *g* for 10 min, and the organic phase was evaporated under a stream of nitrogen gas. The resulting residue was resuspended in a 200 μL mixture of H_2_O and MeOH (50:50 *v*/*v*), and the suspension was centrifuged again at 16,000× *g* for 5 min. The quantification of Fer-Me and its potential hydrolysis product, Fer, was performed by injecting 20 μL of the processed samples into the HPLC system described below. The CSF was withdrawn using the cisternal puncture method [30]. This method allows the virtually blood-free collection of serial CSF samples (30 µL each) through a single needle stick [31]. The time points (*n* = 4–6, maximum of three 30 µL samples/rat collected) were chosen to allow the physiological CSF volume restoration, collecting a maximum total volume of about 100 µL/rat of CSF during the experimental period. Untreated CSF samples (10 µL) were immediately injected into the HPLC system to quantify Fer and Fer-Me.

The HPLC apparatus was a modular system (LC-10 AD VD model pump and SPD-10A VP model variable wavelength UV–Vis detector; Shimadzu, Kyoto, Japan) completed with an injection valve provided of a 20 μL sample loop (model 7725; Rheodyne, IDEX, Torrance, CA, USA). Separations (room temperature) were carried out on a Force Biphenyl column (150 × 4.6 mm, 5 µm) protected by a guard column filled with the same material (Restek, Milan, Italy). Data were acquired and processed through CLASS-VP Software; version 7.2.1 (Shimadzu Italia, Milan, Italy). The mobile phase was eluted under isocratic conditions (0.8 mL/min) and was constituted by a mixture of acetic acid in H_2_O (0.4% *v*/*v*) and MeOH, whose ratio was 40:60 (*v*/*v*). The UV–Vis detector was set at 320 nm for the detection of Fer-Me, its hydrolysis product Fer, and Caf-Me, which was utilized as an internal standard during blood sample extraction. In these experimental conditions, Fer, Fer-Me, and Caf-Me were eluted with retention times corresponding to 4.1 min, 5.0 min, and 8.9 min, respectively. The chromatographic precision was evaluated by repeated analysis (*n* = 6) of the same sample solutions containing each single compound at a concentration of 50 μM (9.71 µg/mL for Fer, 10.41 µg/mL for Fer-Me) dissolved in a mixture of H_2_O and MeOH (50:50 *v*/*v*) and was represented by RSD values ranging from 0.85 to 0.90 for all the compounds analyzed. To ascertain that CSF and blood components did not interfere with the retention times of Fer-Me, its hydrolysis product Fer, and the internal standard Caf-Me, preliminary analyses on blank CSF and extracted blood samples were performed. For calibration purposes, CSF was simulated using standard aliquots of the balanced solution DPBS, which did not contain calcium and magnesium, supplemented with 0.45 mg/mL of BSA [32,33]. Calibration curves of peak areas in function of concentrations for the analytes in simulated CSF were established in the range of 0.1 to 10 μM for Fer (0.019 to 1.94 μg/mL) and Fer-Me (0.021 to 2.08 μg/mL). These curves showed a linear relationship (*n* = 8, *r* ≥ 0.996, *p* < 0.0001). Recovery experiments were conducted on blood samples comparing the peak areas extracted from blood test samples (analytes concentration of 50 μM, 4 °C, *n* = 4) to those obtained from injecting an equivalent concentration of analytes dissolved in a 50:50 *v*/*v* mixture of H_2_O and MeOH. The average recoveries were approximately 40% for Fer and 58% for Fer-Me. For this reason, the concentrations of these compounds were determined by their peak area ratio with respect to the internal standard (Caf-Me). Calibration curves for Fer and Fer-Me were generated in whole blood at 4 °C with eight different concentrations ranging from 0.5 to 250 μM (0.097 to 48.55 μg/mL for Fer and 0.104 to 52.05 μg/mL for Fer-Me), obtaining a linear relationship (*n* = 8, *r* ≥ 0.994, *p* < 0.001).

#### 2.4.2. Nasal Administration of CS-OA NE

Fer-Me and eugenol were nasally co-administered to rats at the doses of 0.4 mg/kg (about 0.1 mg/rat) and 0.8 mg/kg (about 0.2 mg/rat), respectively, as encapsulated in CS-OA NE (Fer-Me content about 1 mg/mL; eugenol content about 2 mg/mL). A group of four rats anesthetized and laid on their backs (weighing approximately 250 g) received CS-OA NE after being fasted for 12 h. In particular, each rat received 50 μL of CS-OA NE formulation in each nostril. The nasal administration was carried out using a semiautomatic pipette connected to a short polyethylene tubing, which was inserted about 0.6 to 0.7 cm into each nostril. Following the administration, blood samples (100 μL) were collected at predetermined time points, along with CSF samples (30 μL). The blood samples (*n* = 4 for timepoint) were immediately mixed with 200 μL of ice-cold CH_3_CN and then 100 μL of internal standard (100 μM BNZ in CH_3_CN) was added. These samples were centrifuged at 12,500× *g* for 5 min. About 300 μL of the supernatant was then withdrawn and centrifuged again. Finally, 10 μL of the resultant sample was analyzed using HPLC for the quantification of Fer-Me and eugenol.

The HPLC apparatus was the same used for the uptake experiments with RPMI 2650 cells (Section 2.3.3). The chromatograms were displayed at a 320 nm wavelength for the detection of BNZ, whose retention time was 3.9 min. A preliminary analysis performed on blank CSF and extracted blood samples evidenced that their components did not interfere with retention times of Fer-Me, eugenol, and the internal standard BNZ. Calibration curves of peak areas versus concentration in the range 0.5 to 10 μM for Fer-Me (0.10 to 2.08 μg/mL), and eugenol (0.08 to 1.64 μg/mL), obtained dissolving the compounds in simulated CSF, were linear (*n* = 8, *r* ≥ 0.996, *p* < 0.0001). The peak areas derived from the extraction of blood test samples (10 μM) at 4 °C (*n* = 6) were compared to those obtained by the injection of equivalent concentrations of the analytes dissolved in a mixture of H_2_O and CH_3_CN (25:75 *v*/*v*), and the average recoveries of compounds extracted were about 69% for Fer-Me and 98% for eugenol, respectively. Therefore, the concentrations of these compounds determined in whole blood were referred to as the peak area ratio with respect to the internal standard BNZ. Eight different concentrations in rat whole blood at 4 °C, ranging from 0.5 to 50 μM for Fer-Me (0.10 to 10.41 μg/mL) and eugenol (0.08 to 8.21 μg/mL) were used to generate calibration curves in this biological system, which were linear (*n* = 8, *r* ≥ 0.990, *p* < 0.001).

#### 2.4.3. In Vivo Pharmacokinetic Calculations

Fer-Me in vivo half-life (t_1/2_) in the rats’ bloodstream was determined using nonlinear regression (exponential decay) of concentration values quantified at appropriate time intervals after infusion, further validated by applying linear regression to the semi-logarithmic plot. The area under the concentration–time curves (AUC, μg∙mL^−1^∙min) in rat bloodstreams or CSF, following Fer-Me intravenous administration or CA-OA NE nasal administration, were calculated using the trapezoidal method.

Following the nasal administration of CS-OA NE, the absolute bioavailability (F) values of Fer-Me and eugenol were calculated as the ratio of the nasal AUC to the intravenous AUC values measured in the bloodstream, normalized to the respective doses, according to Equation (3) [34]:

(3) $$F=\frac{{\mathrm{AUC}}_{\mathrm{nasal}}}{{\mathrm{AUC}}_{\mathrm{IV}}}\cdot \frac{{\mathrm{dose}}_{\mathrm{IV}}}{{\mathrm{dose}}_{\mathrm{nasal}}}\;$$

GraphPad Prism version 7.0 (GraphPad Software, San Diego, CA, USA) was used for all calculations.

### 2.5. Statistical Analysis

Statistical analyses on MTT assays (Section 2.3.2) in RPMI 2650 cells were performed by two-way analysis of variance (ANOVA), followed by Dunnett’s test of multiple comparisons, and unpaired *t*-test.

Statistical analyses on the time course of Fer-Me and eugenol uptake (Section 2.3.3) in RPMI 2650 cells were performed with multiple *t*-tests per row using the Holm–Sidak statistical analysis method.

GraphPad Prism version 7.0 (GraphPad Software, San Diego, CA, USA) was used for all analyses, setting the significance at *p* < 0.05. G power software (Version 3.1.9.7) was used to perform a power analysis to choose the adequate sample sizes for the in vitro cell studies.

## 3. Results

### 3.1. Characterization of CS-OA NE

#### 3.1.1. FTIR Analysis

**Figure 1:**
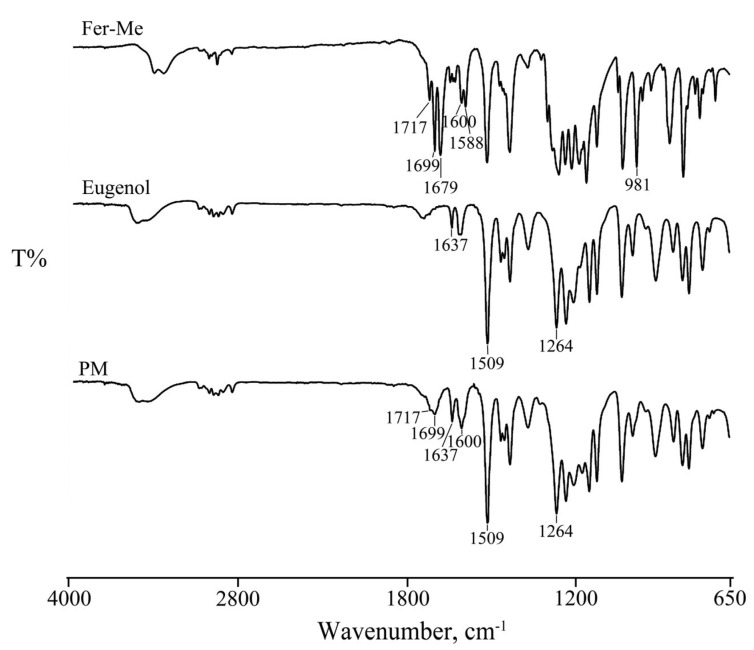
FT-IR spectra of Fer-Me, eugenol, and their physical mixture (PM).

FT-IR spectroscopy was used to evaluate the interactions of Fer-Me in eugenol. The spectra obtained are reported in Figure 1. The stretching and bending bands characteristic of Fer-Me and eugenol functional groups can be clearly identified.

In particular, the spectrum of Fer-Me showed the bands in the region between 3500–3400 cm^−1^ due to the OH stretching vibration. Other bands were evident at 1717–1700 cm^−1^ related to the carbonyl (C=O) group stretching vibration, at 1600, 1588, and 1512 cm^−1^ due to the stretching characteristic of the aromatic ring, and at 981 cm^−1^ related to CH bending.

As reported in the literature [35], the FT-IR spectrum of eugenol, in addition to the bands in the region between 3500–3400 cm^−1^ due to the OH stretching vibration, showed its characteristic peaks at 1637 and 1509 cm^−1^ due to the aromatic C-C stretching, important for its identification, and at 1264 cm^−1^ due to the C-O stretching vibration of the hydroxyl-carbon bound.

The spectrum of their mixture (PM) was from the sum of the two pure components, confirming the homogeneity of the system. The bands characteristic of both actives and the stretching of the aromatic ring were preserved, revealing the absence of significant chemical transformations, supporting the hypothesis that Fer-Me is solubilized in eugenol primarily through physical interactions rather than chemical modifications.

#### 3.1.2. Particle Size Characterization

The particle size analysis on six batches of NEs resulted in an average size of 249.22 ± 32.78 nm and a PDI of 0.24 ± 0.05 nm, indicating consistent formulation with good uniformity.

**Figure 2:**
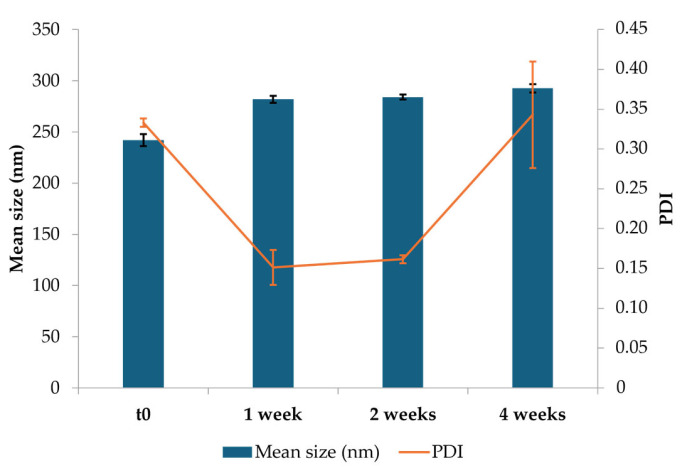
Results of the stability study: average particle size, polydispersity index (PDI), and relative standard deviations for optimized CS-OA NE formulations at the initial time (t_0_), and during storage at 4 °C (mean ± SD).

NEs were stored at 4 °C for four weeks to evaluate the physical stability of the developed formulation. The mean size of three batches of NEs was analyzed after preparation (t_0_) and after 1, 2, and 4 weeks of storage. Observing the graph in Figure 2, NEs exhibited an initial mean particle size of approximately 242 nm (t_0_) with a PDI of 0.33, indicating good uniformity. After one week, it was possible to notice a slight increase in particle size to 282 nm, accompanied by a decrease in PDI to 0.15, suggesting an improvement in size homogeneity, likely due to initial stabilization or reorganization of CS-OA micelles. From the second week onwards, the particle size was maintained constantly, revealing an average size of approximately 293 nm, which was recorded by the third week. In spite of a slight increase up to 0.34, PDI was maintained below 0.5 for all storage times, indicating good homogeneity.

#### 3.1.3. Transmission Electron Microscopy (TEM) Analysis

**Figure 3:**
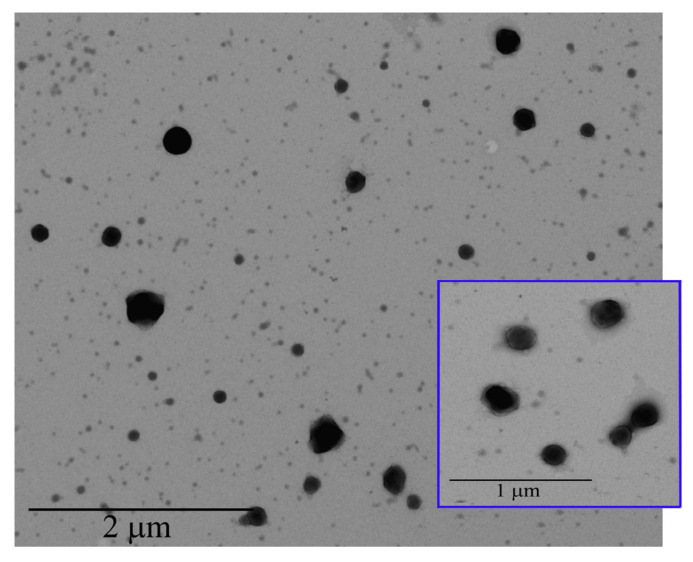
TEM images of CS-OA NE.

TEM analysis was performed to characterize, in terms of morphology, the CS-OA NE formulations (Figure 3). The internal phase of the NE appeared as spherical droplets with an average size of 250 nm, confirming PCS analysis data. In the higher magnification it is possible to observe a shell around the droplets due to the presence of surfactant in the o/w interface.

#### 3.1.4. Process Yield and Encapsulation Efficiency

The NE process yield was evaluated to determine the ability to incorporate and retain active compounds. For Fer-Me, the final concentration was found to be 123.0 ± 0.2 µg per 100 µL of NE, with a % Yield of 97.82 ± 9.77%. In the case of eugenol, the final concentration reached 260.0 ± 0.5 µg per 100 µL of NE, with a % Yield of 67.0 ± 0.2%, indicating a satisfactory ability of the system to encapsulate this compound, which may be influenced by the volatility of the essential oil and its loss during the sample preparation, especially during solvent evaporation.

**Figure 4:**
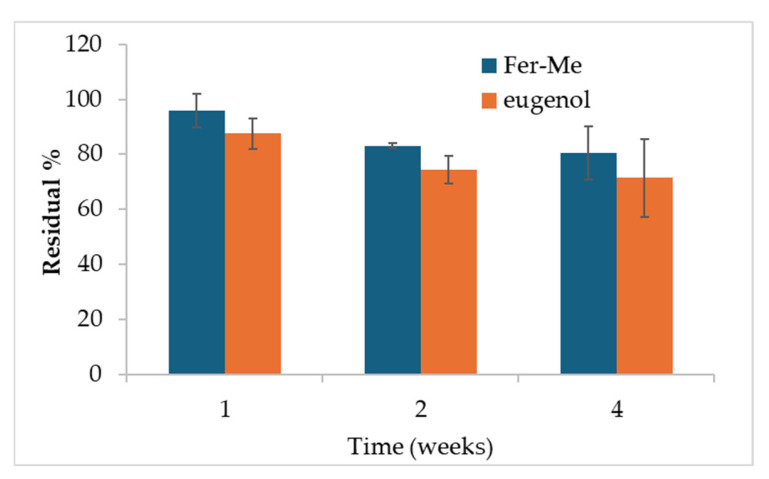
Results of the residual % content of Fer-Me and eugenol after 1, 2, and 4 weeks of storage at 4 °C (mean ± SD). Statistical significance was found for the Fer-Me after 4 weeks vs. 1 week only (ANOVA and Fisher’s least significant difference (LSD) procedure, *p* < 0.05).

In the perspective of the following studies, and especially of cell culture and in vivo studies, a preliminary stability study of the Fer-Me and eugenol contents in the NE samples was performed, maintaining the samples in the refrigerator. As illustrated in Figure 4, a loss of eugenol can be observed especially during the first week probably due to a combination of oxidation and volatility, after which no statistically significant differences were found between 1 and 4 weeks of storage, also due to the variability between the samples.

After 4 weeks, the remaining percentage of eugenol accounts for about 71% (± 14%). This is in line with results obtained in a recent paper for similar formulations of clove oil, of which eugenol is a main component [19]. The CS-OA shell around the hydrophobic core of the droplets stabilizes molecules that are volatile and subject to oxidation, ensuring protection within the formulation [19].

After 4 weeks, the remaining amount of Fer-Me was about 81% (± 10%) with respect to its concentration at time zero, demonstrating that the stability of both Fer-Me and eugenol in the NE was suitable to perform preliminary in vivo studies, although stabilization strategies, such as freeze-drying, should be envisaged for longer storage periods. Encouraging results concerning the possibility of using freeze-drying, maintaining good resuspension of NE, were previously obtained with similar systems [17].

The EE% calculated by HPLC analyses was found to be 90.2% (±0.5%) and 87.9% (±0.7%) for Fer-Me and eugenol, respectively.

#### 3.1.5. Drug Release

**Figure 5:**
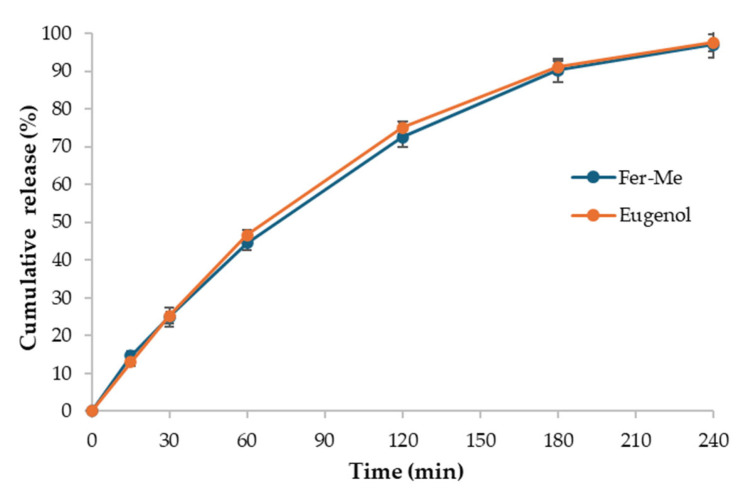
In vitro release of Fer-Me and eugenol from CS-OA NE (mean ± standard deviation, *n* = 3).

The in vitro release studies of Fer-Me and eugenol from CS-OA NE were carried out using the dialysis bag method (Figure 5). The release profiles of Fer-Me and eugenol show a progressive and nearly complete release over time. Both compounds exhibit a rapid release in the first 30 min, with Fer-Me reaching 24.9% (± 2.5%) and eugenol 25.2% (± 2.2%). The release continues to increase steadily until reaching 96.9% (± 3.5%) and 97.5% (± 2.2%) by 240 min for Fer-Me and eugenol, respectively. Overall, the CS-OA NE formulation provides a release compatible with nasal administration.

#### 3.1.6. Mucoadhesion Test

For the zeta potential analysis, different samples were prepared to assess the interaction between mucin and CS in the formulation. The variation in zeta potential provides valuable information regarding the presence of free amine groups on CS, which are positively charged, and their saturation in the presence of mucin. Mucin, with its sialic acid groups, can interact with the protonated amine groups of CS, leading to changes in the surface charge. The results showed a clear shift in the zeta potential values with the addition of mucin. The CS-OA NE exhibited a positive zeta potential of 46.22 ± 0.18 mV that was neutralized (−0.01 ± 0.11 mV) by the interaction with mucin at 0.1% (*w*/*v*) in a 1:1 (*v*/*v*) ratio. When the concentration of mucin was increased to 0.5% (*w*/*v*), the zeta potential further shifted to −21.30 ± 2.12 mV, indicating a further interaction with mucin that completely surrounded the NE, which led to a more pronounced reduction in the positive charge. This shift in zeta potential is consistent with the saturation of CS’s amine groups by mucin’s sialic acid residues, confirming the mucoadhesive potential of the formulation.

### 3.2. In Vitro Cell Studies

#### 3.2.1. Evaluation of CS-OA NE or Fer-Me/Eugenol Solution Toxicity on RPMI 2650 Cells

**Figure 6:**
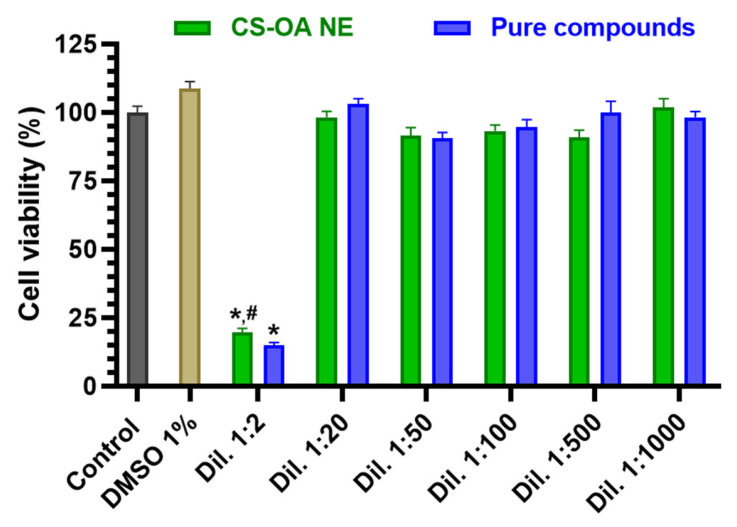
Cell viability of RPMI 2650 cells incubated with different dilutions of CS-OA NE or the corresponding combined solutions of pure compounds (starting from an approximated concentration of 5 mM Fer-Me and 12 mM eugenol in the not-diluted formulation). Data of MTT assay are presented as cell viability percentage (%) normalized to control (in the absence of compounds) and expressed as mean ± S.E.M. of three independent experiments run in duplicate (*n* = 6). Statistical comparisons vs. control were obtained using two-way ANOVA followed by Dunnett’s multiple comparisons test (* *p* < 0.0001 vs. control). Statistical comparisons within the same dilutions were performed by unpaired *t*-test (# *p* < 0.05 CS-OA NE vs. pure compounds).

RPMI 2650 cells were incubated with different dilutions of CS-OA NE (1:2, 1:20, 1:50, 1:100, 1:500, 1:1000 *v*/*v*) or the corresponding combined solutions of Fer-Me and eugenol as pure compounds (whose highest concentrations were 2.5 mM for Fer-Me and 6 mM for eugenol). As shown in Figure 6, both CS-OA NE diluted 1:2 *v*/*v* and the corresponding Fer-Me/eugenol solution reduced the cell viability compared to the control (*p* < 0.0001).

In contrast, no cytotoxic effects were observed for the subsequent dilutions of both CS-OA NE formulation and combined solutions of pure compounds (maximum DMSO concentration = 1%). The absence of toxicity induced by 1% DMSO on RPMI 2650 cells appears in line with previous results obtained in several cell types [36,37].

Considering this result, CS-OA NE was subsequently used for RPMI 2650 cell uptake assay at 1:20 *v*/*v* dilution. The results were then compared to those obtained by incubating RPMI 2650 cells with a solution containing Fer-Me/eugenol at the same concentrations as CS-OA NE.

#### 3.2.2. Evaluation of CS-OA NE or Fer-Me/Eugenol Uptake by RPMI 2650 Cells

RPMI 2650 cells were incubated with (i) CS-OA NE diluted 1:20 *v*/*v* in culture medium, (ii) a combined solution of Fer-Me and eugenol in culture medium at 250 µM (52.05 µg/mL) and 600 µM (98.55 µg/mL), respectively, or (iii) an equivalent amount of raw Fer-Me added directly in the well. Incubations were performed for 10, 30, and 60 min at 37 °C.

**Figure 7:**
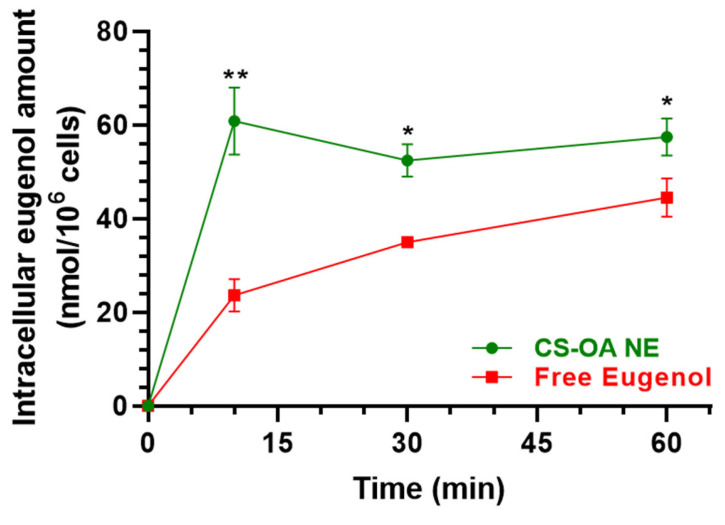
Time-dependent intracellular eugenol amount (nmol/10^6^ cells) in RPMI 2650 cells after incubation with CS-OA NE formulation (green, diluted 1:20 *v*/*v*) or pure eugenol as a combined solution with Fer-Me (red, 600 µM eugenol, 250 µM Fer-Me) in complete A-MEM at 37 °C. Comparisons between CS-OA NE and pure eugenol were performed at each time point with multiple *t*-tests per row using the Holm–Sidak statistical analysis method. Data are expressed as mean ± S.E.M. of four independent experiments. * *p* < 0.05, ** *p* < 0.001.

Figure 7 reports the intracellular uptake profiles in the RPMI 2650 cells of eugenol incubated as loaded in CS-OA NE formulation or as a pure compound in a combined solution with Fer-Me. CS-OA NE allowed us to immediately reach an intracellular eugenol amount of 60.80 ± 5.09 nmol/10^6^ cells, a value that became stable within 60 min (57.41 ± 2.80 nmol/10^6^ cells). On the other hand, when eugenol was incubated as a free compound, its intracellular amount was 23.61 ± 2.45 nmol/10^6^ cells after 10 min and increased to 44.49 ± 2.91 nmol/10^6^ cells within 60 min, indicating a time-dependent eugenol uptake. At all tested time points, the intracellular amounts of eugenol following the pure compound incubation were significantly lower than those obtained following the incubation of CS-OA NE. These results indicate that CS-OA NE formulation allows the improvement of the eugenol uptake in RPMI 2650 cells, suggesting its potential ability to act as a permeability enhancer for this compound across the nasal mucosa. To support this hypothesis, CS-OA NE allowed us to obtain an uptake efficiency at 10 min of 0.77 ± 0.10%, whereas the uptake efficiency of free eugenol at 10 min was 0.26 ± 0.03%, a value which is about three times lower than that of CS-OA NE (*p* < 0.01).

**Figure 8:**
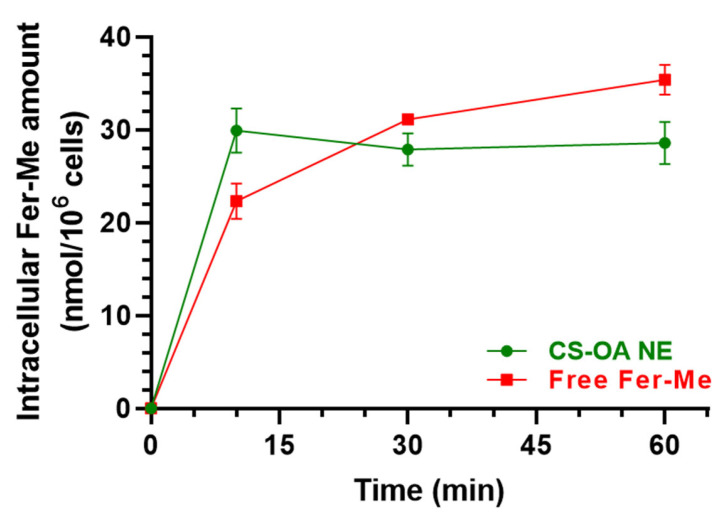
Time-dependent Fer-Me uptake (nmol/10^6^ cells) in RPMI 2650 cells after incubation with CS-OA NE formulation (green, diluted 1:20 *v*/*v*) or pure Fer-Me as a combined solution with eugenol (red, 250 µM Fer-Me, 600 µM eugenol) in complete A-MEM at 37 °C. Comparisons between CS-OA NE and pure Fer-Me were performed at each time point with multiple *t*-tests per row using the Holm–Sidak statistical analysis method, and no significant statistical differences were obtained. Data are expressed as mean ± S.E.M. of four independent experiments.

Figure 8 reports the intracellular uptake of Fer-Me incubated as loaded in CS-OA NE formulation or as a pure compound in a combined solution with eugenol. CS-OA NE allowed Fer-Me to immediately reach an intracellular amount of 29.93 ± 2.37 nmol/10^6^ cells that became stable within 60 min (28.58 ± 2.29 nmol/10^6^ cells), evidencing a profile qualitatively similar to that obtained for eugenol. When Fer-Me was incubated as a free compound, its intracellular amount increased quite slowly during this time (10 min = 22.30 ± 1.93 nmol/10^6^ cells; 60 min = 35.39 ± 1.61 nmol/10^6^ cells). Even though under these experimental conditions a Fer-Me time-dependent uptake could be described, no statistical differences in Fer-Me intracellular amount were found at any time point following the cell incubation with CS-OA NE or Fer-Me/eugenol solution. This trend is confirmed by the uptake efficiencies related to CS-OA NE, whose value was calculated as 0.80 ± 0.12% at 10 min, which was not statistically different from the 0.60 ± 0.05% value obtained for free Fer-Me. Interestingly, no Fer-Me uptake by RPMI 2650 cells was observed following the incubation of an equivalent amount of compound raw powder, directly added to the cell medium in the absence of eugenol, with a consequent inexistent uptake efficiency.

### 3.3. In Vivo Studies

#### 3.3.1. Intravenous Administration of Fer-Me

**Figure 9:**
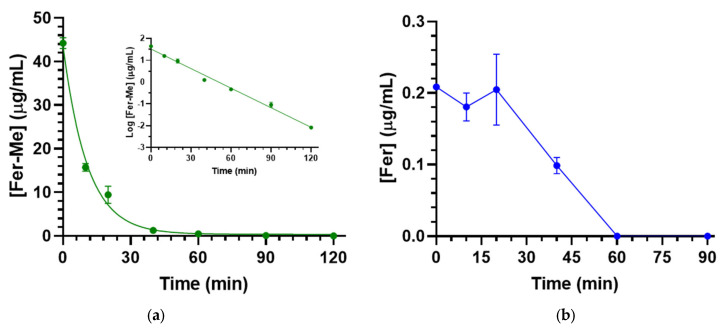
(**a**) Fer-Me elimination profile in rat bloodstreams assessed following the infusion of 2.5 mg of Fer-Me (10 mg/kg) to rats. The elimination process followed apparent first-order kinetics, further evidenced by the semilogarithmic plot shown in the inset (*n* = 7, *r* = 0.993, *p* < 0.0001). Fer-Me half-life was 10.08 ± 0.37 min; (**b**) concentrations of Fer (µg/mL) quantified in rat bloodstreams after the intravenous infusion of 2.5 mg of Fer-Me. Data are presented as the mean ± S.E.M. from four independent experiments.

At the end of Fer-Me intravenous infusion (2.5 mg/rat, i.e., 10 mg/kg dose), the rat plasma concentration of the compound was 44.3 ± 1.3 μg/mL (Table 1). The Fer-Me plasmatic concentration decreased over time (Figure 9a) following apparent first-order kinetics. This was confirmed by the linearity observed in the semilogarithmic plot shown in the inset of Figure 9a (*n* = 7, *r* = 0.993, *p* < 0.0001). The half-life of Fer-Me (t_1/2_) was calculated to be 10.08 ± 0.37 min (Table 1), derived from the kinetic elimination constant (k_el_) value of 0.069 ± 0.003 min^−1^. Additionally, the area under the time/concentration curve (AUC) was determined to be 671 ± 34 μg∙mL^−1^∙min (Table 1).

Due to the possible Fer-Me hydrolysis, rat plasma Fer concentrations were measured following Fer-Me intravenous infusion. As reported in Figure 9b, a small amount (0.209 ± 0.001 µg/mL) of Fer was detected in the rats’ bloodstreams at the end of Fer-Me infusion (Table 1); this concentration remained quite stable for about 20 min (0.205 ± 0.050 µg/mL) and then decreased to zero within 60 min from the end of the infusion. The total Fer AUC value was 8.4 ± 0.8 µg∙mL^−1^∙min (Table 1), corresponding to 1.3% of the Fer-Me AUC value, suggesting that, in vivo, the Fer-Me elimination rate from the rat bloodstream is not significantly lower than its hydrolysis rate.

As a term of comparison, Table 1 reports pharmacokinetic data of Fer and eugenol, previously obtained in our laboratories [11,20], evidencing that the elimination rate of Fer-Me is about two times higher than those of Fer and eugenol; moreover, the plasma concentrations of Fer and Fer-Me appear significantly higher than those of eugenol, by normalizing the doses.

**Figure 10:**
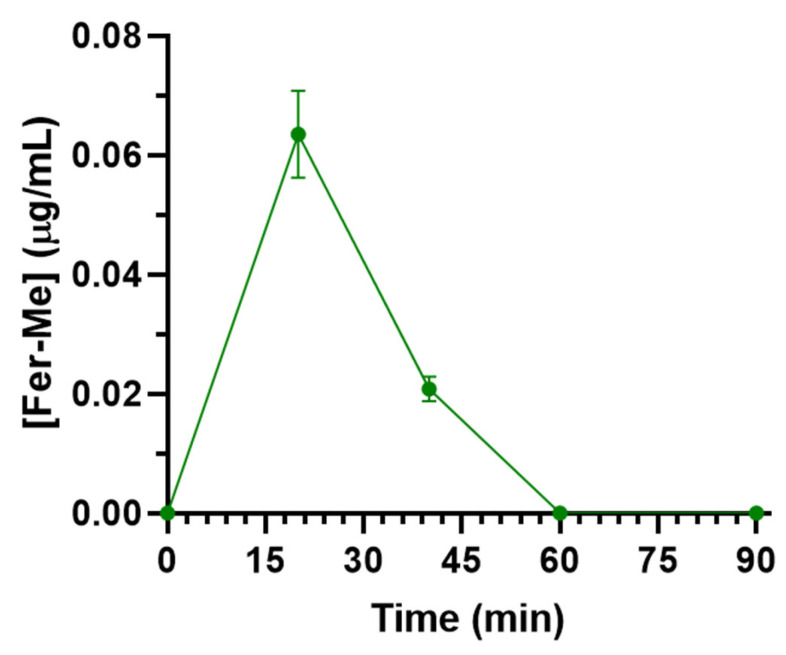
Concentrations of Fer-Me (µg/mL) quantified in rat CSF after the intravenous infusion of 2.5 mg of Fer-Me (10 mg/kg dose). Data are presented as the mean ± S.E.M. from four independent experiments.

Fer-Me rat CSF concentration following intravenous administration was 0.064 ± 0.007 µg/mL 20 min after the end of infusion and then decreased to zero within 60 min (Figure 10). The total AUC value was 1.69 ± 0.15 µg∙mL^−1^∙min (Table 1). The comparison with the pharmacokinetic data of Fer and eugenol in the CSF of rats [11,20], reported in Table 1, indicates that the aptitude of Fer-Me to permeate into the CNS from the bloodstream is poor in comparison to Fer and negligible in comparison to eugenol.

#### 3.3.2. Nasal Administration of CS-OA NE

**Figure 11:**
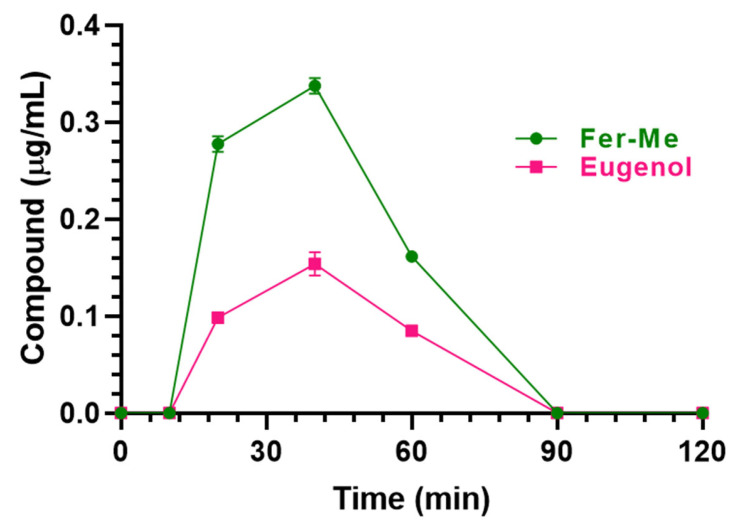
Fer-Me and eugenol concentrations (µg/mL) detected in the bloodstream of rats after nasal administration of a dose of 0.1 mg of Fer-Me (0.4 mg/kg) and 0.2 mg of eugenol (0.8 mg/kg) loaded in CS-OA NE formulation. Data are expressed as the mean ± S.E.M. of four independent experiments.

The nasal administration of Fer-Me (0.4 mg/kg) and eugenol (0.8 mg/kg) loaded in CS-OA NE allowed the compound detection in both rat bloodstreams and CSF. In particular, as reported in Figure 11, C_max_ values of 0.34 ± 0.01 µg/mL and 0.15 ± 0.01 µg/mL were detected in the bloodstream for Fer-Me and eugenol, respectively, 40 min (T_max_) after the CS-OA NE nasal administration (Table 2); thereafter, Fer-Me and eugenol plasma concentrations decreased to zero within 90 min from the administration. The AUC values were 14.95 ± 0.23 µg∙mL^−1^∙min and 6.68 ± 0.27 µg∙mL^−1^∙min for Fer-Me and eugenol, respectively (Table 2). According to these values, the absolute bioavailability values (F) of Fer-Me and eugenol, following CS-OA NE nasal administration, were 56% and 95%, respectively.

**Figure 12:**
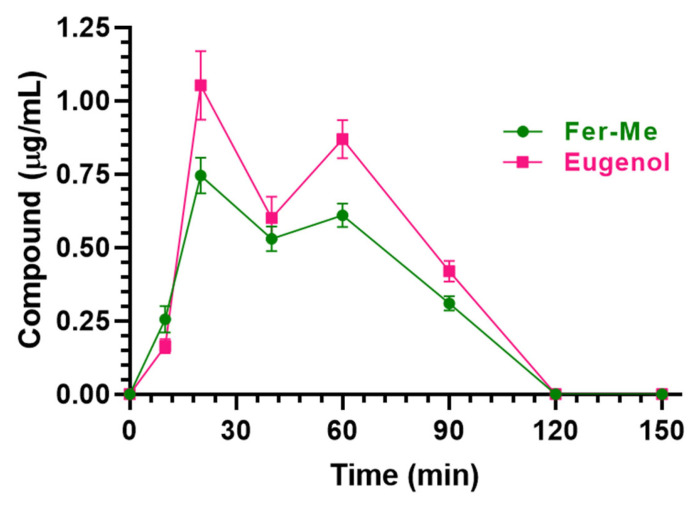
Fer-Me and eugenol concentrations (µg/mL) detected in the CSF of rats after nasal administration of a dose of 0.1 mg of Fer-Me (0.4 mg/kg) and 0.2 mg of eugenol (0.8 mg/kg) loaded in CS-OA NE formulation. Data are expressed as the mean ± S.E.M. of four independent experiments.

As reported in Figure 12, detectable amounts of Fer-Me and eugenol were found in rat CSF following CS-OA NE nasal administration. In particular, the CSF Fer-Me and eugenol concentrations increased up to 0.75 ± 0.04 μg/mL and 1.05 ± 0.08 µg/mL (C_max_), respectively, within 20 min (T_max_) (Table 2) and decreased to zero at 120 min after CS-OA NE nasal administration.

The AUC values were 48.89 ± 1.30 µg∙mL^−1^∙min and 63.75 ± 2.18 µg∙mL^−1^∙min for Fer-Me and eugenol, respectively (Table 2).

It is worth noting that in the present in vivo experiments, a low inter-subject variability was observed for both eugenol and Fer-Me with coefficients of variation (CV%) for C_max_ and AUC parameters of up to 25%.

## 4. Discussion

It is currently recognized that Fer and eugenol, as many other phytochemicals, possess neuroprotective properties, mainly due to their anti-inflammatory and antioxidative effects [1,2,4,6]. Moreover, it has been recently reported that eugenol induces DA release in vitro [11], a property that suggests its possible usefulness in PD treatment and/or prevention. It can be, therefore, hypothesized that the co-administration of the two compounds might be a useful therapeutic strategy against neurodegeneration, especially in PD. However, low CNS bioavailability and/or other pharmacokinetic limitations restrict this potential therapeutic usefulness of Fer and eugenol, especially following their oral or parenteral administration. To possibly overcome this issue, innovative formulations and/or alternative administration routes might be helpful. In this regard, CS-coated polycaprolactone nanoparticles were characterized for nose-to-brain delivery of eugenol [38]. In the present study we describe the design and the characterization of a novel formulation suitable for the nasal Fer-Me and eugenol co-administration, with the final aim to improve the brain targeting of the two phytochemicals, thus possibly maximizing their combined beneficial effects on CNS.

Formulative problems are often associated with the administration of the main components of essential oils, due to their aptitude to separate from aqueous medium; in particular, emulsified or suspended systems obtained with these compounds evidence very poor physical stability [39]. We have previously demonstrated that it is possible to induce spontaneous emulsification processes of the main components of essential oils, such as lemongrass or geraniol, in the presence of the amphiphilic polymer CS-OA, used as a surfactant, generating NEs characterized by satisfactory physical stability [17,18]. Moreover, the nasal administration of this type of emulsion loaded with geraniol allowed us to detect its relevant presence in the CSF of rats, confirming their ability to induce the targeting of the neuroactive agent in the CNS [18]. For these reasons we propose a new emulsified formulation based on eugenol encapsulated in CS-OA salt; eugenol is here designed as a promising vehicle for the active Fer, being able to induce the DA release from dopaminergic neurons and to protect them, together with Fer, from ROS neurodegenerative effects. In the aim of optimizing the active loading in this emulsified formulation, Fer-Me was selected instead of Fer, taking into account that the methyl esterification allows us to increase the encapsulation of Fer in lipid particulate systems [9]. Moreover, Fer-Me evidences antioxidant and anti-inflammatory properties on neuronal cells very similar to those of Fer [9]. The use of CS-OA in this nasal formulation allows us to combine the advantages of CS as a mucoadhesive and absorption enhancer [40] with the stabilization effect it has on the eugenol dispersion [17,18].

The NEs were prepared by the spontaneous emulsification method in the presence of EtOH. The spectrophotometric characterization did not reveal significant chemical interactions between the two components (Fer-Me and eugenol), confirming the homogeneity of the systems in which Fer-Me is solubilized in the essential oil.

The physical stability of the NE has been studied by storing them at 4 °C for 4 weeks. Despite an initial slight increase in particle size, this latter stabilized around 290 nm for the rest of the storage period. The PDI remained below 0.5 during all the tested times, indicating an acceptable dimensional dispersion and thus the ability of CS-OA to stabilize the systems. Moreover, in the perspective of in vivo studies, the % yield of the CS-OA NE was evaluated during the time, evidencing that, after 4 weeks of storage at 4 °C, the stability of both Fer-Me and eugenol was essentially maintained, revealing their remaining amounts ranging between about 70 and 80%. The EE revealed that almost 90% of both compounds are encapsulated into the internal phase of the droplets, revealing also that this type of NE formulation confirms itself as a promising delivery systems with a satisfactory ability to encapsulate and protect actives which can even be volatiles, such as eugenol.

In vitro release profiles revealed a fast initial release of both compounds (about 50% by 60 min) that was almost complete within 4 h. In addition, the use of CS-OA as a stabilizing agent allowed us to obtain a formulation with good mucoadhesive properties that could be exploited for nasal delivery.

The contents of Fer-Me and eugenol in CS-OA NE used for cellular and in vivo studies were about 5 mM and 12 mM, respectively.

To initially characterize the CS-OA NE, the possible cytotoxicity of different dilutions of this formulation has been evaluated in RPMI 2650 cells, which were chosen as an in vitro model of respiratory (i.e., nasal) mucosa. The results evidence that 1:2 *v*/*v* dilution of the CS-OA NE induced cytotoxic effects in RPMI 2650, whereas further dilutions (starting from 1:20 *v*/*v*) were not cytotoxic. It could be hypothesized that the 1:2 *v*/*v* dilution-induced cell death is due to possible cell contamination. However, it is worth noting that the incubation period of the compounds and formulation was 1 h. This short period minimizes the probability that contamination could be the cause of the observed reduction in cell viability at the lowest dilution (1:2 *v*/*v*). Furthermore, eugenol is also recognized as an anti-microbial, antifungal, and antiviral agent [41], and its presence in the sample should contribute to reducing the possibility of cell contamination. Other kinds of NE were previously evidenced to induce cytotoxicity on RPMI 2650 cells, when not appropriately diluted. This effect was attributed to the excessive concentration of the systems’ ingredients in the culture medium [42]. In the present study, this is confirmed by the evidence that a similar cytotoxic effect was observed when Fer-Me and eugenol were added as a pure compound solution to the cell culture medium at similar concentrations to those present in 1:2 *v*/*v* dilution of the CS-OA NE. Similarly to the present results, we recently evidenced that a CS-OA NE containing clove essential oil (whose main component is eugenol) completely maintained the viability of RPMI 2650 cells when it was diluted at least 1:20 *v*/*v* in culture medium, whereas it was toxic at higher concentrations [19]. Concerning this aspect, eugenol may be hypothesized as the main component of the formulation inducing the cytotoxic effects. Indeed, 5 mM eugenol is known to induce cytotoxicity in several cell lines with mechanisms ascribed both to disruption of the structure of mitochondrial and plasma membranes evidenced by mitochondrial membrane potential assay and LDH release assay [43]. It is important to remark that our experimental approach with RPMI 2650 cells can be limited in comparison to air–liquid interface cultured RPMI 2650 cell layers, which produce a mucus barrier in vitro as it is in vivo, therefore representing a more complete pharmacological nasal barrier model for drug permeation studies [44]. We can hypothesize that the present cytotoxic results might be overestimated by the simplified in vitro model used, whereas in vivo mucociliary clearance and mucus produced in in vitro 3D cell models might reduce the cytotoxic effects of the formulation. Further in vivo experiments, also including post-administration histological analysis, are necessary to evaluate whether the mucus layer is able to protect nasal mucosa cells from the CS-OA NE-induced cytotoxicity. This step will obviously be fundamental to understanding the clinical applicability of this formulation strategy.

An improved uptake by RPMI 2650 cells appears crucial for the therapeutic efficacy of drugs [45]. We therefore measured Fer-Me and eugenol intracellular uptake following the incubation of RPMI 2650 cells with CS-OA NE or a Fer-Me/eugenol solution. The uptake studies evidence the ability of CS-OA NE to enhance the intracellular eugenol, but not Fer-Me, amounts during the time, when compared to a Fer-Me/eugenol solution. On the other hand, the incubation of RPMI 2650 cells with solid Fer-Me did not allow the compound cellular uptake. This result appears in good agreement with the previously evidenced low dissolution rate of raw Fer-Me in physiological media [9], further suggesting the relevance of appropriate nasal formulations to induce the permeation across nasal mucosa. In this case, the presence of eugenol can be crucial for the Fer-Me permeability [46]. Based on these encouraging results, the efficacy of the CS-OA NE to induce Fer-Me and eugenol brain targeting was evaluated by the nasal administration of the NE to rats. The administered doses of Fer-Me (0.4 mg/kg) and eugenol (0.8 mg/kg) are in the order of magnitude of the doses of several neuroactive agents previously administered by the nasal route in our laboratories [20,27,28,29]. The pharmacokinetic profiles of eugenol and Fer-Me in the bloodstream and CSF were evaluated and compared with those obtained following their intravenous administration in rats. We previously reported the pharmacokinetic profile of eugenol following its intravenous administration at a dose of 20 mg/kg ([11]; Table 1). This dose, despite being 25 times higher than the one intranasally administered in the present study, did not induce evident side effects [11,47]. To be consistent with this previous experiment, in the present study we chose to intravenously infuse a dose of Fer-Me (10 mg/kg), which was 25 times higher than that intranasally administered (0.4 mg/kg), also considering that no side effects were observed in rats following the administration of Fer at doses up to 40 mg/kg [5].

The aptitude of eugenol to reach the brain following its administration in rats has been properly recognized and appears higher than that of Fer ([11,20]; Table 1). The present study demonstrates that the ability of eugenol to permeate into the brain from the bloodstream is also extremely higher than that of Fer-Me. Indeed, following the intravenous eugenol (20 mg/kg) administration in rats, the CSF AUC was 56.1 ± 4.2 µg·mL^−1^·min [11], i.e., a value that, considering the dose normalization, is about 16 times higher than that obtained following the administration of Fer-Me (10 mg/kg; present study). The evidence that the capability of Fer-Me to reach significant CSF concentrations following its intravenous administration is poor in comparison with that of Fer suggests that Fer-Me may be a substrate of an active efflux transporter (AET) expressed by the blood–brain (BBB) or blood–CSF (BCSFB) barriers. It is, in fact, known that, after lipidization, neuroactive agents can become substrates of this type of transporter [48]. Data reported in Table 1 also indicate that, following the Fer-Me intravenous infusion, its half-life in the bloodstream (about 10 min) is shorter than those of eugenol and Fer (about 20 min) [20]. The short half-life of Fer-Me and its inability to significantly target the brain indicates the poor suitability of this compound to induce clinically relevant CNS therapeutic effects when systemically administered. The very poor ability of Fer-Me to permeate in the CNS from the bloodstream appears to be overcome through the nasal administration of CS-OA NE. This administration strategy, in fact, allows us to obtain surprisingly high absolute plasmatic bioavailability values for either Fer-Me (56%) or eugenol (95%), denoting the ability of the CS-OA NE to act as a permeability enhancer across the nasal mucosa. More importantly, after the nasal administration of CS-OA NE, relevant amounts of Fer-Me were detected in the CSF. According to the respective AUC values, the CSF bioavailability of Fer-Me following the nasal administration of CS-OA NE was, indeed, about 30 times higher than that obtained after the intravenous administration of a 25 times higher dose of the compound (Table 2). We have previously demonstrated that Fer ranging from 0.5 μM to 40 μM is able to induce anti-inflammatory effects on neuronal cells treated with muramyl dipeptide. This effect was also evidenced for the Fer derivative obtained by its ester conjugation with Fer-Me (Fer-Fer-Me, a prodrug of both Fer and Fer-Me) [20]. Taking into account that, in the present study, the Fer-Me concentrations in the CSF of rats ranged between 0.26 μg/mL (1.3 μM) and 0.75 μg/mL (3.6 μM) after the nasal administration of the CS-OA NE (Figure 12), we may hypothesize that these values are suitable to induce anti-inflammatory effects at a central level, similarly to Fer or Fer-Fer-Me. Concerning eugenol, the nasal administration of CS-OA NE allowed us to obtain, in the rat CSF, similar amounts to those reached following the intravenous administration of a 25-times-higher dose of the compound. It is worth noting that eugenol oral administration is associated with a poor absolute bioavailability (about 4% [11]), which likely offsets its great ability to permeate into the CNS from the bloodstream. An oral dose of 500 mg/kg of eugenol was indeed necessary to obtain in the CSF of rats an AUC value of 30.97 ± 2.18 μg·mL^−1^·min [11], which is about half with respect to that obtained by the nasal administration of a 600-times-lower dose of eugenol loaded in the CS-OA NE. The nasal administration of eugenol may be therefore considered an interesting alternative to its oral administration, aimed at improving the compound bioavailability. Moreover, after the nasal administration of CS-OA NE, the eugenol permanence in the rat CSF was longer (120 min) than those observed following eugenol intravenous or oral administration (50 min; [11]). We have previously evidenced that a significant increase in DA release can be obtained from neuronal differentiated PC12 cells following the incubation with eugenol at concentrations ranging from 0.5 μM (0.08 μg/mL) to 25 μM (4 μg/mL). In particular, the highest release amounts were obtained within 15 min after the treatment with eugenol concentrations within 1 μM (0.16 μg/mL) [11]. As reported in Figure 12, the eugenol concentration obtained in the rat CSF was 0.16 μg/mL (1 μM) 10 min after the CS-OA NE nasal administration, then the eugenol concentrations ranged between 1.05 μg/mL (6.6 μM) and 0.42 μg/mL (2.6 μM) within 90 min. These values seem therefore suitable to induce DA release at a central level, according to our previous in vitro data obtained with neuronal cells.

It is worth noting that in the present in vivo experiments, inter-subject variability of all pharmacokinetic parameters (CV% range = 2–25%) was very low for both eugenol and Fer-Me. Although this could be due to the maximum efforts to standardize the present animal experiment conditions, it can be speculated that these compound pharmacokinetic properties are promising for an easily controllable therapy in humans. Nonetheless, it remains to be investigated whether this predictability will also hold in clinical studies.

Overall, the present data suggest that the CS-OA NE nasal formulation may be a promising drug delivery system to induce the CNS targeting of Fer-Me and eugenol, and to prolong their permanence in the brain, thus possibly maximizing their beneficial effects against neurodegenerative diseases. It seems likely that CS-OA NE mucoadhesive or permeation-enhancing effects play a fundamental role in the observed increase in compound brain bioavailability after the formulation’s nasal administration. Concerning this aspect, we have previously observed that NE of clove oil (whose main component is eugenol) stabilized with CS-OA evidence mucoadhesive properties which are properly attributed to a CS shell localized at the surface of the essential oil nano-droplets [17,19]. Taking into account that the ability of CS to open tight junctions in the olfactory and respiratory nasal epithelia is recognized [40], it may be reasonable to hypothesize that the CS coating of the dispersed droplets of the emulsion can induce a permeation enhancing effect. Further experiments are required to confirm this hypothesis.

## 5. Conclusions and Perspectives

The high ROS production derived from the elevated basal energy metabolism of SNc dopaminergic neurons has been recently proposed as an important cause of the vulnerability of cells, with the consequent striatal DA loss that characterizes PD [13,14]. Currently, the loss of DA in PD patients is mainly compensated by the L-DOPA administration which causes, in the CNS, a DA excess whose metabolism produces further ROS and, therefore, further neurodegeneration [49,50]. Inhibitors of DA-catabolizing enzymes can allow us to reduce the L-DOPA doses but, similarly to other anti-PD drugs, do not arrest the progression of neurodegenerative processes [51]. The stimulation of DA release by dopaminergic neurons jointed to their protection from oxidative stress may be, therefore, a promising approach for the prevention and therapy of PD. The co-administration of Fer and eugenol, by combining the neuroprotective properties of both compounds [1,2,4,6] with the DA-releasing effects of eugenol [11], might be suitable to this purpose. The ability to easily reach the brain from the bloodstream characterizes both eugenol [11] and Fer [5,20,52,53]. A relatively high oral bioavailability was also evidenced for Fer [54,55] but, on the other hand, its fast elimination rate from the body does not allow its adequate permanence in the brain for therapeutic proposals [53,56]. Similar negative aspects characterize eugenol, whose very poor oral bioavailability and fast elimination from the body reduce the opportunity to obtain prolonged therapeutic effects in the brain, following oral administration [11]. The nasal formulation based on CS-OA NE designed for the co-administration of a Fer derivative (Fer-Me) and a main component of essential oil (eugenol), proposed in the present study, might contribute to overcoming Fer and eugenol pharmacokinetic issues, by increasing the compound brain targeting and permanence. The jointed presence of these compounds in the brain of PD patients may therefore protect the dopaminergic neurons from neurodegeneration, inducing them, at the same time, to release the DA necessary to contrast the motor impairment. Further experiments on preclinical PD models are necessary to validate this hypothesis.

## Figures and Tables

**Table 1:** Intravenous administration of Fer, Fer-Me, or eugenol to rats. Half-life (t_1/2_) and concentrations at the end of intravenous infusion (C_0_) in the bloodstream are reported. The administration of Fer-Me allowed for the detection in the rat bloodstream of its hydrolysis product, Fer, whose C_0_ value is also reported. The area under the concentration–time curve (AUC) values are provided for all compounds studied. Additionally, the highest concentrations (C_max_), their corresponding times (T_max_), and the AUC values obtained for all compounds in cerebrospinal fluid (CSF) are included. Data are reported as the mean ± S.E.M. from four independent experiments. The values referred to Fer and eugenol were previously obtained in our laboratories and published; the references are indicated in the table.

Bloodstream					
Administration (Dose)	Compound	C_o_ (μg·mL^−1^)	t_1/2_ (Min)	AUC (μg·mL^−1^·min)	Reference
Fer (1 mg/kg)	Fer	10.5 ± 1.0	20.3 ± 1.3	244 ± 13	[20]
Fer-Me (10 mg/kg)	Fer-Me	44.3 ± 1.3	10.08 ± 0.37	671 ± 34	Current data
	Fer	0.209 ± 0.001	-	8.4 ± 0.8	Current data
Eugenol (20 mg/kg)	Eugenol	16.5 ± 0.2	19.4 ± 2.1	174.8 ± 3.1	[11]
**CSF**					
**Administration (Dose)**	**Compound**	**C_max_** **(μg·mL^−1^)**	**T_max_** **(Min)**	**AUC** **(μg·mL^−1^·min)**	**Reference**
Fer (1 mg/kg)	Fer	0.08 ± 0.01	60	3.3 ± 0.3	[20]
Fer-Me (10 mg/kg)	Fer-Me	0.064 ± 0.007	20	1.69 ± 0.15	Current data
Eugenol (20 mg/kg)	Eugenol	2.79 ± 0.18	10	56.1 ± 4.2	[11]

**Table 2:** Nasal administration of CS-OA NE to rats, corresponding to 0.1 mg of Fer-Me (0.4 mg/kg) and 0.2 mg of eugenol (0.8 mg/kg). The highest concentrations (C_max_), their corresponding times (T_max_), and the AUC values obtained for Fer-Me and eugenol in rat bloodstreams and cerebrospinal fluid (CSF) are included. Fer-Me and eugenol absolute bioavailability (F) refers solely to the bloodstream. Data are presented as the mean ± S.E.M. from four independent experiments.

Bloodstream				
Compound (Dose)	C_max_ (μg·mL^−1^)	T_max_ (Min)	AUC (μg·mL^−1^·min)	F (%)
Fer-Me (0.4 mg/kg)	0.34 ± 0.01	40	14.95 ± 0.23	56
Eugenol (0.8 mg/kg)	0.15 ± 0.01	40	6.68 ± 0.27	95
**CSF**				
**Compound** **(Dose)**	**C_max_** **(μg·mL^−1^)**	**T_max_** **(Min)**	**AUC** **(μg·mL^−1^·min)**	**-**
Fer-Me (0.4 mg/kg)	0.75 ± 0.04	20	48.89 ± 1.30	-
Eugenol (0.8 mg/kg)	1.05 ± 0.08	20	63.75 ± 2.18	-

## Data Availability

The original contributions presented in this study are included in the article. Further inquiries can be directed to the corresponding author.
